# Hydrated Salt/Graphite/Polyelectrolyte Organic-Inorganic Hybrids for Efficient Thermochemical Storage

**DOI:** 10.3390/nano9030420

**Published:** 2019-03-12

**Authors:** Sergio Salviati, Federico Carosio, Guido Saracco, Alberto Fina

**Affiliations:** 1 Dipartimento di Scienza Applicata e Tecnologia, Politecnico di Torino-Alessandria Campus, 15121 Alessandria, Italy; sergio.salviati@polito.it (S.S.); guido.saracco@polito.it (G.S.); alberto.fina@polito.it (A.F.); 2 Center for Sustainable Future Technologies, Istituto Italiano di Tecnologia, 10144 Torino, Italy

**Keywords:** thermal energy storage, thermochemical energy storage, salt hydration, composite materials, heat transfer, mass transfer

## Abstract

Hydrated salt thermochemical energy storage (TES) is a promising technology for high density energy storage, in principle opening the way for applications in seasonal storage. However, severe limitations are affecting large scale applications, related to their poor thermal and mechanical stability on hydration/dehydration cycling. In this paper, we report the preparation and characterization of composite materials manufactured with a wet impregnation method using strontium bromide hexahydrate (SBH) as a thermochemical storage material, combined with expanded natural graphite (G). In addition to these fully inorganic formulations, an organic polyelectrolyte (PDAC, polydiallyldimethylammonium chloride) was exploited in the structure, with the aim to stabilize the salt, while contributing to the sorption/desorption process. Different formulations were prepared with varying PDAC concentration to study its contribution to material morphology, by electron microscopy and X-ray diffraction, as well as water sorption/desorption properties, by thermogravimetry and differential calorimetry. Furthermore, the SBH/G/PDAC powder mixture was pressed to form tabs that were analyzed in a climatic chamber, which is evidence for an active role of PDAC in the improvement of water sorption, coupled with a significant enhancement of mechanical resistance upon hydration/dehydration cycling. Therefore, the addition of the polyelectrolyte is proposed as an innovative approach in the fabrication of efficient and durable TES devices.

## 1. Introduction

Fighting climate change is one of the biggest challenges, attracting research efforts from all over the world. Smart heat management is presently a central topic in greenhouse gas mitigation and the approach of thermal energy storage (TES) has a key role in achieving this goal [1]. The simple and fundamental concept at the base of TES is to identify heat sources with low environmental and economic impact, store their energy when it is not needed, and use it in later times instead of producing it with other traditional alternatives. These technologies have been proven to have a potential reduction in CO_2_ emissions up to 5.5% compared to 1990 levels [2]. In particular, low temperature heat sources (up to 150 °C) are considered among the greatest opportunities in this field. These sources can be identified mainly in two scenarios: Waste heat reuse and smart heat management in buildings. In the first case, low grade heat that is commonly released to the environment may in principle be conveniently recovered and stored for later use. Several sources were identified in different industrial fields such as oil, chemicals, steel, glass [3], and food [4] industries, as well as municipal solid waste, mining wastes [5], data centers [6], and car engines [7]. On the other hand, one of the main issues in energy management in the building sector is the mismatch between heat supply and demand. In this field, two main areas are identified: Short-term storage (e.g., from night to day), and long-term storage (e.g., from summer to winter). Following these needs, many TES devices were successfully developed and implemented in both residential and commercial structures [8]. In addition to this, the increasing development of renewable energy sources creates the need to reinvent energy demand management resulting in integrated solutions where TES technologies are coupled with photovoltaic panels and/or solar thermal technologies in order to manage the peak of electricity demand and reduce the costs related to electricity consumption [9,10].

The most used classification of TES materials takes into account the form in which heat is stored. Sensible heat storage is the most widely adopted and well-known techniques, because it is based on cheap and highly available materials (e.g., water or concrete) with high specific heat. The second approach uses the latent heat of phase change materials (PCMs), such as paraffins, to obtain higher energy storage densities with respect to sensible heat [11]. The third class is thermochemical TES and it includes materials showing a high-enthalpy reversible gas/solid reaction. One of the most promising thermochemical materials (TCMs) class is inorganic salt hydrates (M_n_A_m_∙XH_2_O) [12], in which the storage reaction is:

M_n_A_m_∙XH_2_O + heat ↔ M_n_A_m_∙(X − Y)H_2_O + YH_2_O

When heat is transferred from a selected source to the TCM, in the so-defined charging step, dehydration occurs. As long as the salt is maintained in the dehydrated state, latent heat is stored. When water is made available to the salt (discharging step), hydration occurs and hydration heat is released. The possibility to control the heat release by controlling the water feed to the dehydrated salt, is one of the main advantages of this technique, making the heat discharge controllable on demand [13]. The second important advantage of TCMs over PCMs is the higher (one order of magnitude) energy storage density associated with the employed materials [14]. Due to this great potential, many efforts were made to identify the best performing salt hydrates with both experimental [15,16], and theoretical methods [17,18]. Despite the selection of the hydrated salt primarily depending on the available source temperature, one of the most promising TCMs discussed in literature for low temperature TES applications is SrBr_2_∙6H_2_O (SBH) owing to its effective combination of a relatively high storage density (798 kJ/kg) and low dehydration temperature (≃100 °C) as reviewed in a detailed study [19]. Unfortunately, first efforts to implement this salt in a working device also showed some severe limitations, in terms of low thermal conductivity, low chemical stability over hydration/dehydration cycles, and slow mass/heat transfer [20]. Research efforts were mostly aimed at overcoming these limits by design improvement, while few studies dealt with new material concepts in order to fully exploit the potentialities of SBH. One practical approach is to include the TCMs in a porous matrix to overcome the drawbacks of solid/gas reactions [21]. In particular, expanded natural graphite (G) was proposed for combination with TCMs, based on its low price and density, coupled with high thermal conductivity and surface area. Recently, hybrid salt/graphite materials were prepared and tested [22,23,24], but in most cases an inherent incompatibility between the structures of the two materials resulted in big salt aggregates, thus minimizing water adsorption kinetics, and the rate of heat transfer between salt and graphite layers. In this manuscript, we aim at overcoming these limitations by producing graphite composites encompassing a polyelectrolyte binder, PDAC (polydiallyldimethylammonium chloride), to enhance the compatibility between salt and matrix. Indeed, PDAC is known to have a strong interaction with graphite layers [25], and it is expected to show a good affinity with ionic materials due to its high charge density. The aim is to obtain a better distribution of salt on the pores surfaces, maximizing the area of the air/salt and salt/graphite interfaces. In addition, PDAC also shows good moisture sorption ability, thus potentially improving the hydration kinetics of the salt [26].

## 2. Experimental Section

### 2.1. Materials

PDAC (Mw = 400,000–500,000 g/mol) was purchased from Sigma-Aldrich^®^ as 20% wt/wt water solution. In order to obtain solid PDAC samples for the analyses, the solution was dried in an oven at 120 °C until constant weight was reached and hydrated in a climatic chamber at 23 °C and 50% relative humidity (RH) overnight. Expanded natural graphite (G), with 28.4 m^2^/g surface area (as reported in the material datasheet) was purchased by TIMCAL (Bodio, Switzerland), commercial grade TIMREX^®^ BNB90. SrBr_2_·6H_2_O (S) with >95% purity in powder form was purchased from Alfa Aesar^®^ (Haverhill, MA, US). All reagents were used as received for preparing stable water dispersions using deionized water supplied by a Direct-Q^®^ 3 UV Millipore System (Milano, Italy).

### 2.2. TCM Composite Manufacturing

**Figure 1:**
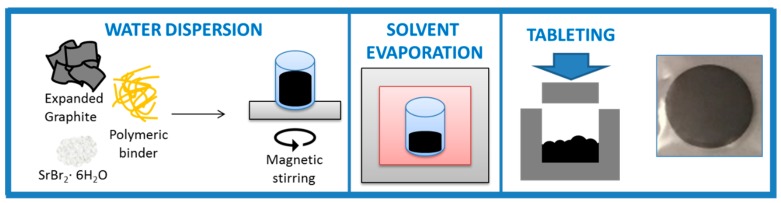
Thermochemical material (TCM) composite material manufacturing process.

The main steps in the manufacturing process are depicted in Figure 1.

PDAC water solution was diluted with 30 mL of water with subsequent additions of G and SBH. Four samples were prepared varying the amount of polymer while keeping the G/SBH ratio constant (their compositions are shown in Table 1).

The suspension was stirred overnight to obtain a homogeneous dispersion and then heated at 100 °C on a plate while stirring for around 5 h to remove water via evaporation. In this step, SBH and PDAC started precipitating on the graphite matrix. When the dispersion viscosity was too high to allow any further stirring, the wet material was placed in a vacuum oven at 50 °C overnight to complete the process. After these steps, the prepared mixtures were subjected to a complete dehydration and hydration cycle prior to the subsequent charge/discharge cycles [27]. The samples were dehydrated in a ventilated oven at 120 °C until they reached constant weight and rehydrated in a climatic chamber at 23 °C and 50% RH. After that the samples were tableted using a stainless steel mold in a hydraulic press under the pressure of 1 t. The nominal tab size was 30 mm in diameter and 3 mm in height.

### 2.3. Characterization

The materials morphology was investigated with a LEO-1450VP (Zeiss, Oberkochen, Germany) scanning electron microscope (SEM) with a 15 kV accelerating voltage, on the cross section of tabs, obtained by fragile fracture upon bending. Surfaces were gold-coated prior to SEM observations.

XRD analyses were performed on a Philips/Panalytical X´Pert Pro (Malvern, Milano, Italy) using a Philips PW3040/60 X-ray generator with a Cu anode using a Kα wavelength. A broad interval of 2θ angles of 10–70 were chosen to identify the SBH structure using a 0.026° 2θ as scan step and nominal time per step of 100 s, using a scanning PixCell 1d detector. Intensity of reported diffractograms was normalized on an SBH (110) peak.

The performance of the composite materials was investigated with both differential scanning calorimetry (DSC) on a TA Instruments Q20 system (TA Instruments, Milano, Italy) using open aluminum pans and thermogravimetric analysis (TGA) on a TA Instruments Discovery gravimetric balance using open platinum pans. Both experiments were performed with the same temperature program: An equilibration at 35 °C, a heating ramp to 90 °C at 10 °C/min, and an isotherm for 90 min with a dry nitrogen flux of 50 mL/min for DSC and 25 mL/min for TGA. Only for the PDAC sample, was the isotherm time set to 10 h to assure complete dehydration. Samples weight was ≈7 ± 0.5 mg.

Thermal conductivity tests of the prepared tabs were carried out on a TPS 2500S by Hot Disk AB (Göteborg, Sweden) with a Kapton sensor (radius 6.4 mm) using the slab method [28]. Before each measurement, specimens were stored in a constant climate chamber (Binder KBF 240, Tuttlingen, Germany) at 23.0 ± 0.1 °C and 50.0 ± 0.1% RH for at least 48 h before tests. The test temperature (23.00 ± 0.01 °C) was controlled by a silicon oil bath (Haake A40, Thermo Scientific Inc., Waltham, MA USA) equipped with a temperature controller (Haake AC200, Thermo Scientific Inc., Waltham, MA, USA).

A custom setup was assembled to observe the hydration of the prepared composites. The tabs were vertically held to maximize the surface area exposed to the environment. They were dehydrated in an oven at 120 °C until they reached constant weight. After that they were placed in a climatic chamber (Binder KBF 240, D) at 23.0 ± 0.1 °C, and 50.0 ± 0.1% RH, and weighted on an analytical balance (Radwag AS 220.R2, PL) with an accuracy of ±0.5 mg to record the hydration over time. An experimental deviation of ±10% on the normalized mass gain during rehydration was estimated, after having performed several tests.

## 3. Results and Discussion

### 3.1. Morphology Analysis

As the microstructure of the TCM may have affected the kinetic of hydration/dehydration, SEM was employed to investigate the influence of PDAC concentration on the morphology and microstructure of the prepared samples.

**Figure 2:**
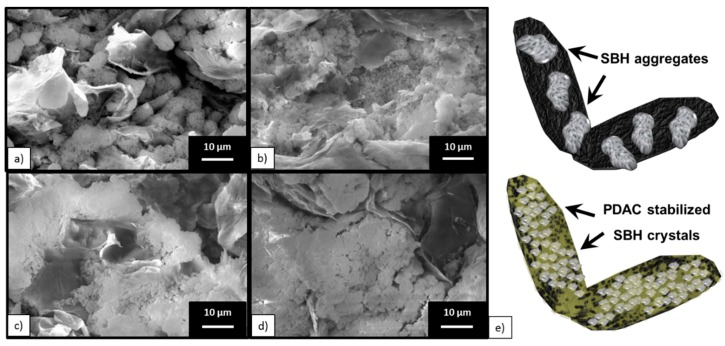
SEM representative images of samples cross sections. (**a**) Strontium bromide hexahydrate/expanded natural graphite (SBH/G), (**b**) SBH/G/P(0.1), (**c**) SBH/G/P(0.5) (**d**) SBH/G/P(1). (**e**) Illustration of the polydiallyldimethylammonium chloride (PDAC) effect in the SBH/G mixture.

**Figure 3:**
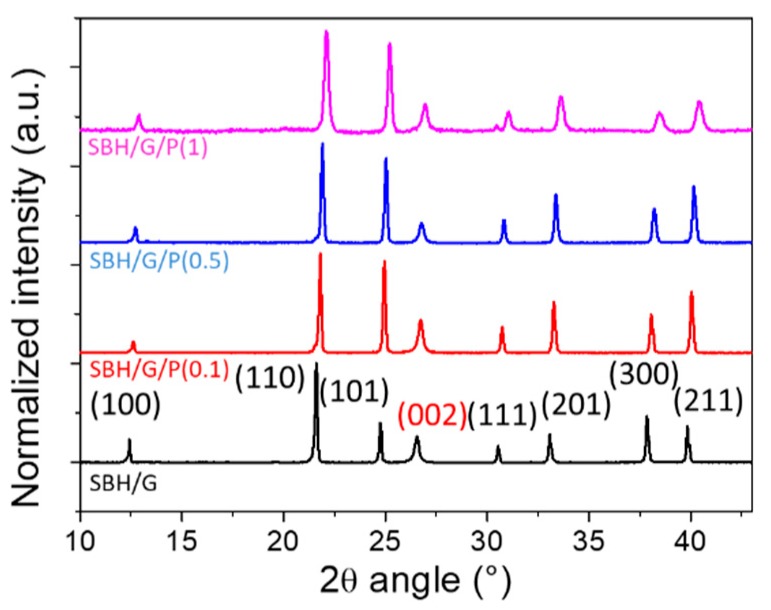
XRD diffractograms of the prepared samples. Miller indices are depicted in black for SBH and in red for graphite crystal planes. Intensity is normalized on the SBH (110) peak.

The micrographs (Figure 2) unveil the effect of PDAC in the salt distribution. In particular, in the absence of PDAC, salt aggregates in globular shapes with dimensions in the order of few μm were observed between flakes of expanded graphite. In the presence of PDAC, the shape of salt aggregation changes evolved with polyelectrolyte concentration. Indeed, the average size of globular salt agglomerates were reduced in the sample with a PDAC/G ratio of 0.1 (PDAC content of 2% w/w, Figure 2b), and completely absent in the samples with ratio 0.5 (PDAC content of 10% w/w of PDAC) and 1 (PDAC content of 18% w/w of PDAC), as shown in Figure 2c,d respectively. It appears that the PDAC acted as a binder between salt crystals as well as an adhesion promoter at the salt/graphite interface, as schematized in Figure 2e. XRD analysis was used to identify the crystal structure of the employed salt, in particular, it was used to investigate possible anion exchange reactions between SBH and the polyelectrolyte during the water dissolution and recrystallization process. Diffraction patterns for SBH/G and the counterparts with different PDAC concentrations are reported in of Figure 3, while the collected diffractograms for purchased SBH and G are reported in Figure S1.

Using the Joint Committee on Powder Diffraction Standards-International Centre for Diffraction Data database (JCPDS-ICDD) [29], both SrBr_2_∙6H_2_O and graphite crystal planes were identified, their Miller indices being reported on the diffractogram in black and red, respectively. The absence of additional peaks excluded the formation of crystalline byproducts derived from ion exchanges between PDAC and SBH during the manufacturing process. Nonetheless, differences in relative intensities of selected peaks (i.e., signals at 24.7° and 39.8°) were observable in the diffractogram between samples with and without polyelectrolyte addition. This could be ascribed to the influence of the polyelectrolyte in the growth of salt hydrates crystals, as previously reported in the literature [30]. In addition, a limited broadening of the SBH main peaks was observed at the highest PDAC concentration, confirming the binder role in the aggregation of SBH crystals. Finally, the absence of extra peaks in PDAC-containing composites confirmed the amorphous nature of the polyelectrolyte.

### 3.2. Thermal Properties

**Figure 4:**
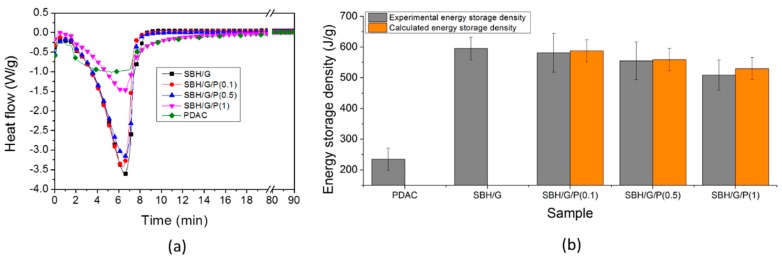
(**a**) Differential scanning calorimetry (DSC) curves of the prepared samples. (**b**) Experimental and calculated values for energy storage density.

DSC and TGA analyses were first employed to study the dehydration of the composites in dry conditions. The temperature program was chosen to simulate a 90 °C heat source charging the thermochemical system. DSC results are reported in Figure 4, showing heat flow plots for different samples, characterized by an endothermic peak corresponding to the dehydration reaction. 

**Figure 5:**
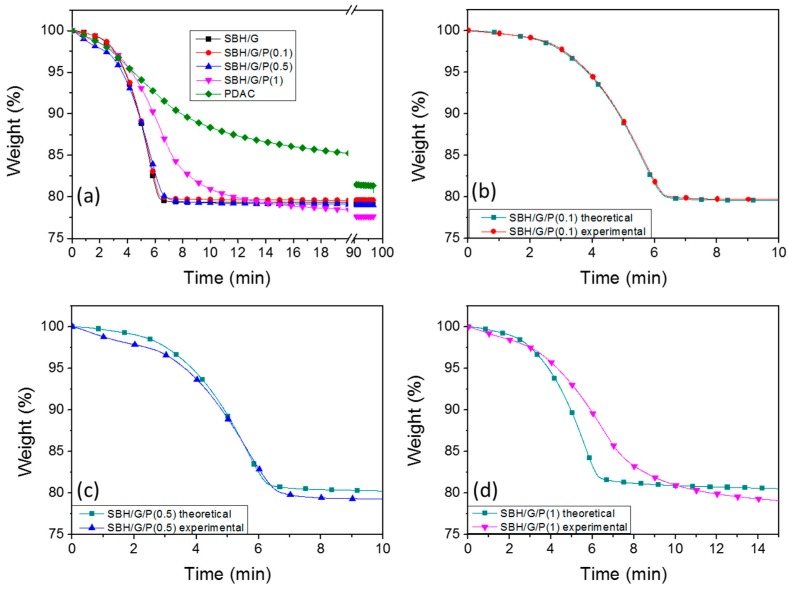
Thermogravimetric analysis (TGA) weight plots of the different SBH/G/PDAC mixtures, compared with pristine PDAC (**a**), and comparison between experimental end calculated data for SBH/G/P(0.1) (**b**), SBH/G/P(0.5) (**c**), and SBH/G/P(1) (**d**).

Figure 4b reports the energy storage density values, calculated from the integral of heat flow plots (average of at least three measurements). For SBH/G/PDAC mixtures, the experimental values were compared with the theoretical values, calculated according with the rule of mixture (Equation (1)), based on the individual components of energy storage density, and their concentration in the mixture.

(1) $$E_{c}=x_{p}E_{p}+x_{s}E_{s}$$

where *E_c_* is the energy density of the composite materials with SBH, G, and PDAC, *E_p_* is the energy density of PDAC, *E_s_* is the energy density of SBH/G and *x_p_* and *x_s_* are the weight fractions of PDAC and SBH/G in the final composites, respectively. As reported in Figure 4b, the experimental values were corresponding to the expected ones, within experimental deviations; this points out that the system acts as an ideal mixture of the two components, with no synergic nor antagonist interaction between PDAC and SBH, in terms of total energy stored. By increasing the content of PDAC, a lowering of the total energy storage density was obtained, diminishing the efficiency of the composite for thermal storage applications by approximately 15% at the highest PDAC concentration. This was ascribed to the great difference in energy storage density between PDAC and the SBH, which was related to the different hydration mechanisms of the two substances. The dehydration of prepared samples were also evaluated by TGA measurements allowing for the assessment of the amount and kinetics of water removal upon heating, as a function of the polyelectrolyte concentration (Figure 5).

At low PDAC concentrations (0.1 and 0.5 PDAC/G weight ratios), the polyelectrolyte did not significantly alter the dehydration kinetics with respect to the SBH/G composite. On the other hand, SBH/G/P(1) exhibited a delayed weight loss compared to SBH/G, reflecting the slow dehydration kinetic observed for PDAC. To further investigate the effect of PDAC, the theoretical weight loss curves (*W_th_*) for the composites were calculated by applying a rule of mixture between the neat PDAC and SBH/G (Equation (2)).

(2) $$W_{c}\left(t\right)=x_{p}W_{p}\left(t\right)+x_{s}W_{s}\left(t\right)$$

where *W_p_* and *x_p_* are the weight and mass fraction of PDAC in the composite, while *W_s_* and *x_s_* are the weight and mass fraction of SBH in the composite, respectively. As reported in Figure 5b,c, the samples with 0.1 or 0.5 weight ratios showed limited differences between the theoretical and experimental plots, thus suggesting two dehydration processes, from PDAC and SBH, to proceed independently. For SBH/G/P(1), a significant deviation was observed between theoretical and experimental plots, suggesting that kinetics of dehydration were controlled by the interaction between the two phases. This is consistent with the polyelectrolyte binding action between the salt crystals, observed by SEM and ascribed to the delayed diffusion of the water, released by the salt, through the polyelectrolyte. Indeed, while the release of water from crystalline hydrated SBH was simply triggered by the temperature, the amorphous structure of PDAC, with its high free volume, broadened the water release in time, through a series of absorption/desorption steps, eventually reduced the overall dehydration rate. The above results suggest that a high concentration of polyelectrolyte binder can partially reduce the efficiency of the thermochemical system under study by both decreasing the heat storage density (Figure 4b) and slowing down water dehydration kinetics (Figure 5d). Thus, only composites with a PDAC/G weight ratio of 0.1 and 0.5 were selected for tablet preparation and hydration kinetics characterization.

### 3.3. Composite Tabs Hydration

**Figure 6:**
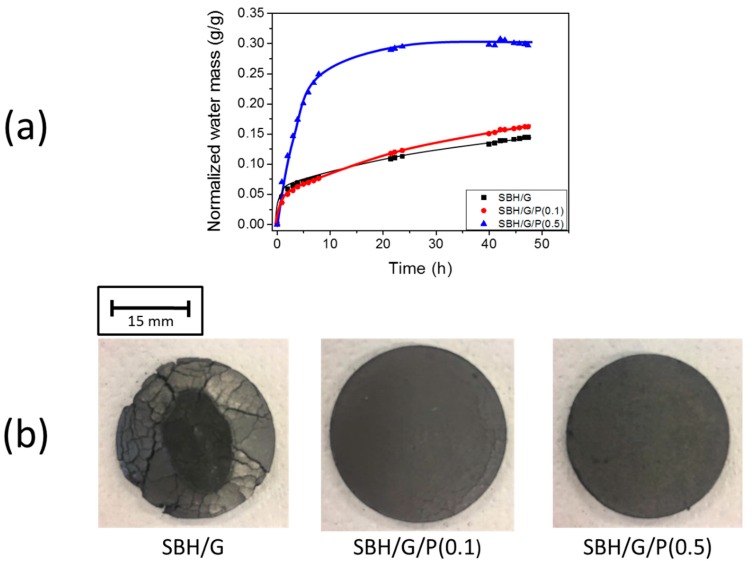
(**a**) Hydration test on composites tabs; (**b**) pictures of the tabs after one dehydration/hydration cycle.

It is known that the surface/volume ratio can strongly influence the heat and mass transfer phenomena in a thermochemical storage system [23]. For this reason, while values collected with dry TGA and DSC analyses may be used to compare the performance of different composites, these are not representative of a real application, both in terms of mass and moisture effects. In order to obtain more realistic results, hydration kinetics have been evaluated in a climatic chamber on composites tabs having dimensions suitable for real applications. Hydration curves, calculated as the measured weight gain normalized over the weight of the dry tabs, are reported in Figure 6a.

The plots clearly show a monotonic weight gain for both SBH/G and counterparts including PDAC. However, dramatic differences in moisture absorption kinetics were proven as a function of PDAC concentration. Indeed, while limited differences existed between SBH/G/P(0.1) and SBH/G, the hydration rate of SBH/G/P(0.5) was much higher, especially within the first hours of the test, reaching the full hydration of both phases in the mixture (equivalent to 0.26 g_water_/g_mixture_ calculated on the basis of Equation (2)) within approximately 10 h, whereas hydration of the other samples was still ongoing after 45 h (Figure 6a). Another important aspect to evaluate when considering a real application was represented by the durability of the prepared composite. This had a potentially strong impact on the effectiveness and practicability of the thermochemical storage solution. In the present study we observed severe damage in the form of cracks to the SBH/G tabs immediately after the first hydration/dehydration cycle (Figure 6b), thus proving this aspect to be a significant weakness of the graphite/salt hydrate composite approach. The mechanical stress in the samples may have been caused by the volume change of the salt during the hydration process. In fact, it is reported that the density of strontium bromide changed between 3.5 g/cm^3^ and 2.4 g/cm^3^ from hexahydrate to monohydrate form [31]. On the other hand, the presence of a polymeric binder appeared to reduce crack formation at a polymer/G ratio of 0.1, and completely prevent it a 0.5 ratio, thus maintaining the structural integrity of the composites.

In addition, the thermal conductivity of prepared composite tabs was also evaluated, as heat exchange was obviously crucial for the efficiency of heat storage devices. Results reported in Figure S2 show that the thermal conductivity of the prepared composites remained constant within the experimental error, in the 16–16.5 W/mK range, demonstrating no detrimental effects related to the presence of the polyelectrolyte. Furthermore, thermal conductivity values obtained in this work were significantly higher than previously reported values for similar graphite SBH composites. [24,32]. As clearly depicted by the characterization reported in Figure 6, the composite with a PDAC/G ratio of 0.5 was capable of achieving superior water adsorption kinetics while maintaining high thermal conductivity values thus proving that the inclusion of a polymer binder was a successful strategy for the design of an efficient thermochemical storage solution.

## 4. Conclusions

This work was focused on the production of composites comprising of strontium bromide hexahydrate, expanded natural graphite, and polydiallyldimethylammonium chloride for thermochemical energy storage applications, using a simple and environmentally sustainable, water-based process.

Morphological analysis performed by SEM showed a stabilizing effect of the polymer binder on the salt particles, while XRD data confirmed the presence of SrBr_2_∙6H_2_O in the final material without undesired byproducts. The materials were characterized with different thermal analysis techniques to understand their performance in terms of energy storage density and capability of heat and mass transfer. The prepared composites were further molded in centimeter scale tabs suitable for exploitation in a modular-design reactor, in order to analyze their hydration kinetics and thermal conductivity properties. High contents of PDAC polyelectrolyte (SBH/G/P(1)) resulted in slightly limited dehydration kinetics and energy storage densities.

On the other hand, lower PDAC contents (SBH/G/P(0.1) or SBH/G/P(0.5)) did not affect dehydration kinetics and caused minimal reduction if energy storage density. Tabs prepared with SBH/G/P(0.5) were found to have significantly higher hydration rates in ambient conditions (23 °C and 50% RH) with respect to the conventional SBH/G composites. Indeed, the polyelectrolyte-containing formulation allowed us to reach complete hydration of the tabs in ~10 h, while the samples with no PDAC reached only ~35% of total hydration in the same time. These results relate to the physical action of the organic polyelectrolyte, acting as a binder between salt crystals, controlling moisture diffusion, and mechanically stabilizing the structure against stress-cracking, which is typical of pristine salt and salt/graphite formulations. Furthermore, a state of the art value of 16 W/mK thermal conductivity was obtained for the tabs, almost independent of the presence of the polyelectrolyte.

The results collected in this paper clearly demonstrate the proposed approach as a promising strategy for the design of efficient thermochemical storage solutions. Future studies should aim to investigate the cyclability of multiple hydration/dehydration cycles of the composite tabs, under controlled air flow, as well as their engineering in order to exploit processing conditions and geometries capable of reducing charge/discharge cycles and improving the efficiency of the system. Mechanical characterizations of the tabs might also prove important to better understand the stabilizing effect of the polyelectrolyte in the composite structure. All these characteristics will help in the fabrication of a TCM suitable for low grade heat reuse and ready for a scale up in prototypes focused on specific applications.

## Figures and Tables

**Table 1:** Summary of the prepared samples.

Sample Name	Weight Ratio		
	G	SBH	PDAC
	Expanded Natural Graphite	Strontium Bromide Hexahydrate	Polyelectrolyte Binder
SBH/G	1	5	0
SBH/G/P 0.1)	1	5	0.1
SBH/G/P(0.5)	1	5	0.5
SBH/G/P(1)	1	5	1

## Supplementary Materials

The following are available online at https://www.mdpi.com/2079-4991/9/3/420/s1, Figure S1: Collected XRD diffractograms for (**a**) SrBr_2_∙6H_2_O and, (**b**) expanded natural graphite. Figure S2: Thermal conductivity data of prepared composites.
