# Supplementary material for: Hydrated Salt/Graphite/Polyelectrolyte Organic-Inorganic Hybrids for Efficient Thermochemical Storage
Source: Nanomaterials (Basel). 2019 Mar 12;9(3):420. doi: 10.3390/nano9030420 (PMC6473937; doi:10.3390/nano9030420)
Supplement: Supplementary file 1 [file nanomaterials-09-00420-s001.pdf]

Hydrated Salt/Graphite/Polyelectrolyte organic-inorganic hybrids for thermochemical storage

Sergio Salviati [1,2], Federico Carosio [1,*], Guido Saracco [1,2] and Alberto Fina [1]

1 Dipartimento di Scienza Applicata e Tecnologia, Politecnico di Torino-Alessandria Campus, 15121, Alessandria, Italy;
2 Center for Sustainable Future Technologies, Istituto Italiano di Tecnologia, 10144, Torino, Italy;
* Correspondence: federico.carosio@polito.it

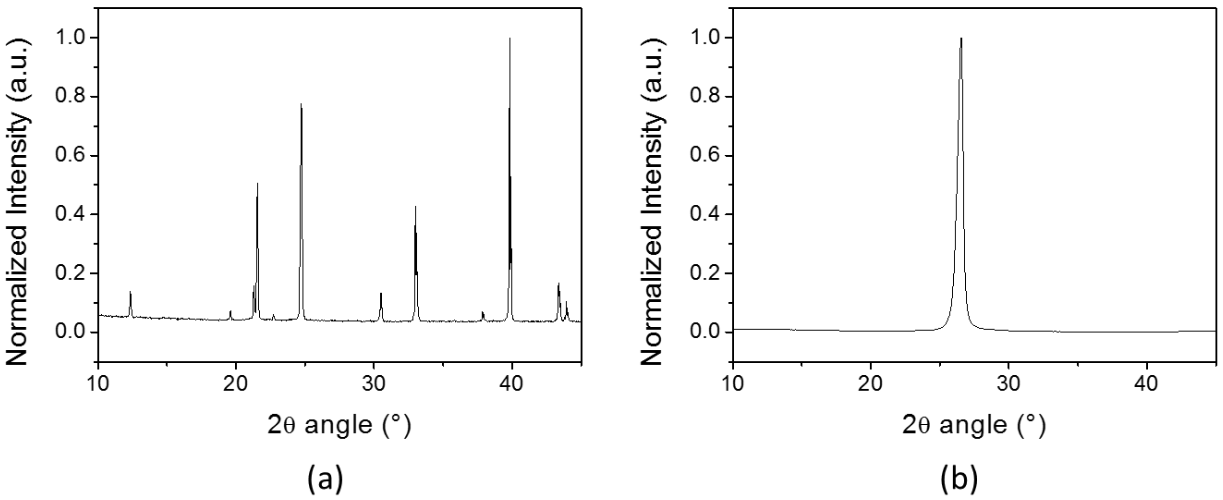

Figure S 1 Collected XRD diffractograms for (a) $SrBr_2{\cdot}6H_2O$ and (b) Expanded Natural Graphite.

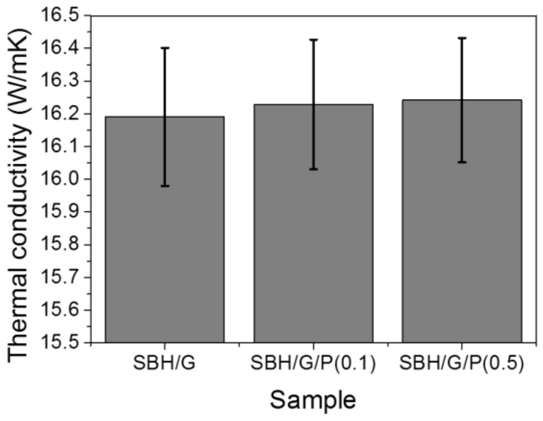

Figure S 2 Collected data on thermal conductivity
